# Dordaviprone in H3K27M-mutant diffuse midline glioma: an editorial on emerging targeted therapy

**DOI:** 10.1097/MS9.0000000000004079

**Published:** 2025-10-13

**Authors:** Saliha Zafar, Muhammad Furqan, Sadia Binte Rahim, Muhammad Usman Haider

**Affiliations:** a Department of Medicine, Foundation Medical College, Islamabad, Pakistan; b Department of Medicine, King Edward Medical University, Lahore, Pakistan; c Department of Medicine, Mymensingh Medical College Hospital, Mymensingh, Bangladesh; d Department of Internal Medicine, Geisinger Health System, Wilkes-Barre, Pennsylvania, USA

**Keywords:** diffuse midline glioma, dordaviprone, DRD2 antagonism, H3K27M mutation, ONC201

## Abstract

Diffuse midline glioma (DMG) with H3K27 mutation is a highly aggressive grade 4 tumor with poor prognosis and limited treatment options. Dordaviprone, a first-in-class oral imipridone, recently received Food and Drug Administration approval for pediatric and adult patients with H3K27M-mutant DMG. In pooled analyses, it demonstrated meaningful clinical activity, with an overall response rate of up to 30% and durable disease control, particularly when administered after radiotherapy. Mechanistically, it targets the dopamine receptor D2 and ClpP, inducing apoptosis through both metabolic and epigenetic pathways. Treatment was well tolerated, with only grade 1–2 adverse events reported. Larger randomized studies are needed to validate efficacy and long-term safety.

Diffuse midline glioma (DMG) with H3K27 mutation is a grade 4 malignant tumor that mainly affects the midline structures in the brain, such as the brainstem, thalamus, and spinal cord. This H3K27 conversion is more usual in pediatric age, and it shows a consistently aggressive clinical course^[1]^. It has been revealed that H3K27 conversion, most importantly, the lysine-to-methionine mutation at position 27 of the H3 histone tail, plays a crucial role in the pathogenesis of DMG^[2]^. These mutations happen to produce epigenetic dysregulations, which will further cause modifications in chromatin structure and dysregulation of gene expression in DMG tumor cells, showing an aggressive tumor behavior^[2]^. DMG has an inferior prognosis, with most patients surviving less than a year after diagnosis, as they are highly invasive, spreading into adjacent brain structures^[3]^. Radiotherapy remains the primary treatment for DMG, providing temporary symptom relief and slowing progression. However, recurrence is almost inevitable, with survival averaging 9–12 months. Surgery is not an option due to the brainstem’s critical anatomy, and no chemotherapy, immunotherapy, or targeted therapy has improved survival, mainly because of poor BBB penetration and tumor resistance^[4]^.

In August 2025, the Food and Drug Administration (FDA) granted clearance for dordaviprone, a first-in-class oral small molecule imipridonean. It is intended for use in pediatric (1 year and older) and adult patients with DMG who have the H3 K27M mutation^[5]^. This approval is based on the results of a pooled analysis of 50 patients across five non-randomized clinical trials conducted in the United States. For children and adolescents, body weight is taken into account when determining the required dose. Patients weighing over 52.5 kg receive the full 625 mg dose, while those weighing less than 52.5 kg receive a reduced dose of approximately 125 mg^[6]^. Dordaviprone functions through a bimodal mechanism, acting as a selective antagonist of the dopamine receptor D2, a G-protein coupled receptor, and as an allosteric activator of the mitochondrial protease ClpP. The coupling of these targets triggers the ATF4/CHOP-driven integrated stress response, leading to the expression of TRAIL and its receptor, DR5. In parallel, dordaviprone reduces oxidative phosphorylation via c-Myc and interferes with Akt/ERK signaling, collectively promoting tumor cell apoptosis. TRAIL/DR5-dependent apoptosis is the dominant outcome; dordaviprone can also trigger parallel apoptotic pathways, cell cycle arrest, or antiproliferative effects^[7]^.

In a pooled analysis of 50 patients (46 adults, 4 children) with recurrent H3 K27M DMG, oral dordaviprone produced an overall response rate (ORR) of 20% as per Response Assessment in Neuro-Oncology (RANO) for high-grade glioma criteria, with one complete response (CR) and nine partial responses (PRs). The disease control rate (DCR; CR + PR + stable disease) was 40%, with a median duration of response of 11.2 months, a time to response of 8.3 months, a 35.1% progression-free survival at 6 months, and a median overall survival of 13.7 months^[8]^. By RANO for low-grade glioma, the ORR was 26% (six PRs, seven minor responses) and the DCR was 42%. When assessed using the best responses from either RANO- high-grade glioma or RANO-low-grade glioma, the ORR was 30% (1 CR, 9 PRs, 5 minor responses) and the DCR was 44%. Corticosteroids and functional (Karnofsky/Lansky Performance Status) response rates showed additional clinical benefit, though less consistently. Subgroup analyses suggested better outcomes in patients with higher performance status, while no responses were observed in those with multiple target lesions^[8]^.

Patients with H3K27M mutant DMG historically lack effective systemic therapies; however, emerging data with dordaviprone indicate meaningful clinical activity, particularly when given after radiotherapy but before recurrence, where median overall survival extended to 21.7 months compared with 9.3 months in the recurrent setting. Radiographic responses were linked to baseline tumor expression of tricarboxylic acid cycle-related genes, suggesting potential molecular predictors of benefit^[9]^. Mechanistically, dordaviprone appears to influence both metabolic and epigenetic pathways, including the elevation of 2-hydroxyglutarate levels and the restoration of H3K27me3, alongside the transcriptional repression of genes involved in cell cycle and neuroglial differentiation. Collectively, these findings support dordaviprone as the first single-agent therapy to demonstrate improved outcomes in this molecularly defined glioma subtype^[9]^.

Treatment-related adverse events were observed in eight patients (66.7%), all grade 1–2, including fatigue (16.7%), and single cases (8.3% each) of headache, nausea, anemia, diarrhea, elevated ALT, hypertension, lymphopenia, increased amylase, ataxia, facial edema, hypophosphatemia, insomnia, and weight gain^[10]^. Four patients (33.3%) developed serious adverse events (AEs) deemed unrelated to therapy, such as dysphagia (16.7%), and isolated cases of vomiting, fatigue, gait disturbance, fever, headache, and hydrocephalus. No treatment-related serious AEs, dose reductions, or dose-limiting toxicity occurred. Only one patient (8.3%) discontinued therapy due to AEs attributed to disease progression rather than the drug^[10]^.

Dordaviprone has shown encouraging early results, but the supporting data come from a relatively small group of patients. This limits the generalizability of the findings. To strengthen the evidence base, larger randomized trials with extended follow-up are necessary to confirm its clinical benefit and capture long-term safety signals. Further work should also address its role in pediatric and diverse populations, evaluate potential drug–drug interactions, and incorporate real-world outcomes and quality-of-life measures. Only through such comprehensive studies can the drug’s actual therapeutic value and safety profile be fully established.

## Data Availability

The datasets are available from the corresponding author on reasonable request.

## Research registration unique identifying number (UIN)

This is not an original research project involving human participants in an interventional or an observational study but a case report. This registration was not required.
